# Brucellosis, the Forgotten Endemic: A Clinical Case Report

**DOI:** 10.7759/cureus.77761

**Published:** 2025-01-21

**Authors:** Ana Dourado, Ângela Rodrigues, Maria Eugénia Afonso, Maria Montanha, Miguel Cardoso, Sara Ervedosa, Viviana Gonçalves, Elisabete Mendes

**Affiliations:** 1 Pathology and Laboratory Medicine, Unidade Local de Saúde do Nordeste, Bragança, PRT; 2 Internal Medicine, Unidade Local de Saúde do Alto Alentejo, Portalegre, PRT

**Keywords:** brucellosis, occupational disease, prevention, serological test, zoonosis

## Abstract

Brucellosis is a disease caused by an intracellular Gram-negative coccobacillus, which presents nonspecific symptoms and is difficult to diagnose. There are several species of the genus *Brucella* currently known involved in its pathogenesis. In Portugal, in the Bragança district, *Brucella melitensis* stands out as an infection agent.

Since the 1990s, diagnostic tests associated with brucellosis have become more regularly part of clinical and laboratory practice. In recent years, several new cases of brucellosis have been identified in humans within the Local Health Unit of the Northeast (ULSNE), its total area of influence being the district of Bragança in Portugal. As for the number of positive serological tests on animals, these have also been high.

The clinical case presented follows a regional pattern, involving a 41-year-old female patient who sought emergency care due to low back pain and weight loss, three to four weeks ago, after ingesting artisanal fresh cheese. The Rose Bengal test was performed and monitored with the Wright reaction, which allowed the diagnosis and respective treatment with double antibiotic therapy to be made. Despite the therapy and measures implemented, the clinical picture evolved unfavorably, with the emergence of new complications.

Due to its non-specific clinic, brucellosis is a current underdiagnosed disease, which is why it is necessary to insist on training and clinical screening of people and animals. It is also essential to support research projects in the field of zoonoses as well as sharing information with other regions or countries that experience these realities, thus allowing for a better understanding and management of these diseases.

## Introduction

Worldwide, approximately 500,000 cases of brucellosis are reported annually and it is estimated that there are 2.4 billion people at risk [1].

According to the 2020 report presented by the European Centre for Disease Prevention and Control (ECDPC), Portugal is the second country in Europe with the most cases of brucellosis [2]. This disease is currently underdiagnosed, in part due to the presence of a nonspecific and insidious clinical presentation. For this reason, as Mendes et al. point out, this is also underreported [3]. Thus, compared with the remaining countries of the European Union (EU), in Portugal, brucellosis maintains the visibility of cases [3]. According to the same ECDPC report, in this country, the incidence rate of reported cases per 100,000 inhabitants was 0.09, with nine diagnoses reported. In the district of Bragança, two cases were diagnosed (0.002 per 100,000 inhabitants). In this context, 197 cases of positive serology for brucellosis in animals were also identified in 2020. The record of serological tests carried out, by municipality, shows a non-uniform diversity of cases [4].

In Portugal, in the Trás-os-Montes region, economic activity is closely linked to livestock production. In this context, direct or indirect interaction between humans and infected animals may be associated with the transmission of several zoonoses, with brucellosis standing out due to its incidence and prevalence. The Trás-os-Montes region is considered an endemic area, having been the scene of debilitating situations over the years. In the 1990s, there was an average of 80 new cases per year, with a higher incidence in men, due to greater exposure to rural work activities.

Brucellosis is caused by Gram-negative coccobacilli-shaped bacteria of the genus *Brucella*. Several species are currently known, the most clinically important being, *Brucella abortus* (cattle), *Brucella suis* (associated with pigs), *Brucella canis* (associated with dogs), and *Brucella melitensis* (associated with sheep and goats). The last one is responsible for the majority of cases recorded in the district of Bragança,because of sheep and goat farming, according to Pappas et al. [5]. *Brucella melitensis* and *suis *have the highest pathogenicity, while *Brucella abortus* and *Brucella canis* have moderate pathogenicity. *Brucella melitensis* causes the most severe cases of brucellosis and is the most prevalent worldwide [1].

The transmission of the disease occurs through the ingestion of undercooked meat or unpasteurized dairy products, through the inhalation of aerosols by slaughterhouse and meatpacking plant employees and laboratory professionals, according to the 2022 National Animal Feed Production Report by the Directorate-General for Food and Veterinary Medicine, Government of Portugal, as well as contact with skin wounds, breastfeeding, and tissue transplants or blood transfusions [4]. After entering cells, the bacteria are transported to the local lymph nodes, where the reproduction process begins, first via the lymphatic route and then via the hematogenous route. The causative bacteria can spread to any organ or system in the body. 

Brucellosis is a systemic disease, whose presentation and duration of the clinical picture allow it to be characterized in acute, subacute, chronic, and localized forms. The incubation period ranges from two to four weeks. Patients usually present non-specific symptoms, namely fever, night sweats, arthralgia, and/or asthenia [6]. Weight loss, headaches, anorexia, abdominal pain, cough, and/or depression may also occur. Hepatomegaly, splenomegaly, and/or lymphadenopathy can be present on physical examination [7]. The complications of the disease are associated with the focus of the infection in various locations, namely at the osteoarticular level. There is a 30% chance of osteoarticular focus [7]. In Europe, most cases of brucellosis require hospitalization (64.5%), resulting in a mortality rate of 3.5% [2].

In the clinical analysis laboratory, diagnosis continues to be made using the Rose Bengal serum agglutination test, which has proven to be an excellent diagnostic method in the acute phase. The Wright test, also referred to as a diagnostic method by some authors, plays a fundamental role in monitoring the progress of treatment through a methodology of successive dilutions that allow us to quantify, in a semi-quantitative way, the antibodies developed and, therefore, the progress of treatment [8]. However, there are other diagnostic options, ranging from measuring antibodies by enzyme-linked immunosorbent assay (ELISA) to ​polymerase chain reaction (PCR) research, which are used less frequently, as they are more expensive and time-consuming. As mentioned in Order No. 1150/2021, of the Official Gazette, most laboratories continue to rely on serodiagnosis due to its low cost and the reliability of the results [9].

The mainstay of treatment for brucellosis is double antibiotic therapy. In patients over eight years of age, doxycycline 100 mg, orally, twice a day, for six weeks and rifampicin 600 to 900 mg, orally, once a day, for six weeks are generally indicated, in uncomplicated cases [10,11]. Other treatment options are doxycycline plus an aminoglycoside (streptomycin or gentamicin) or fluoroquinolone. Doxycycline for six weeks plus streptomycin (or gentamicin) for 14 days reduces the recurrence rate. For uncomplicated cases, rifampicin for six weeks may be used instead of an aminoglycoside. Fluoroquinolone regimens for 14 to 42 days plus rifampicin or doxycycline instead of an aminoglycoside have, in small studies, been shown to be equally effective, but these regimens are not preferred. In children, under eight years of age, the usual treatment is with sulfamethoxazole/trimethoprim plus rifampicin for four to six weeks. Neurobrucellosis and endocarditis require prolonged treatment and three antibiotics are commonly administered [11]. However, the duration of the therapeutic regimen depends on the presence of relapses and the therapeutic scope of the etiological agent in question [10].

Prevention is the best form of combat, avoiding the ingestion of undercooked animal products, the consumption of unpasteurized dairy products or those without quality assurance, and the handling of animal waste without the use of appropriate protective equipment, namely gloves, rubber, and aprons [11].

## Case presentation

**Figure 1:**
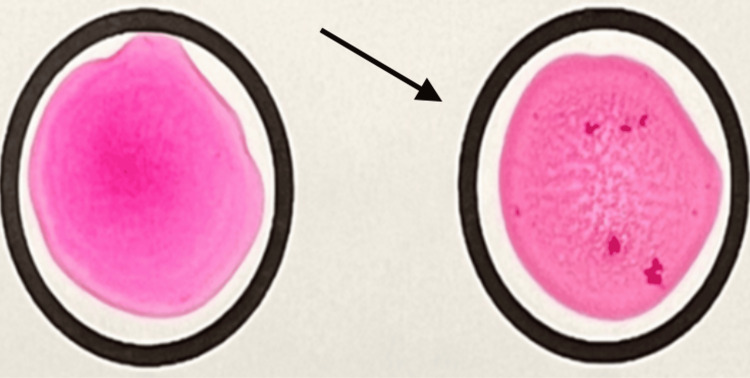
Rose Bengal test. The Rose Bengal test is a rapid screening method whose positive results must be confirmed by other tests. In this context, the figure shows, on the left, a negative test. On the right, an agglutination reaction is evidenced, illustrating a positive Rose Bengal test.

The clinical case presented refers to a 41-year-old female patient, a farmer, without recent trips, autonomous and cognitively integrate. She lived in a rural environment, in a country house, with goat and sheep farming. She had a medical history of type II diabetes mellitus, well controlled with the combination of dapagliflozin and metformin, and active smoking habits. On January 3, 2022, she consulted her General and Family Medicine physician due to low back pain and weight loss. Without any other associated symptoms. She reported consuming unpasteurized dairy products in December 2021, namely unpasteurized cheese. In view of this, some differential diagnoses are considered, such as zoonosis, namely brucellosis, tuberculosis, and/or neoplastic disease. An analytical study was requested, namely inflammatory/infectious parameters, and a Rose Bengal test was performed, with a positive result (Figure 1). The diagnosis of brucellosis was confirmed. In this context, the patient was medicated with doxycycline and rifampicin for three months.

**Figure 2:**
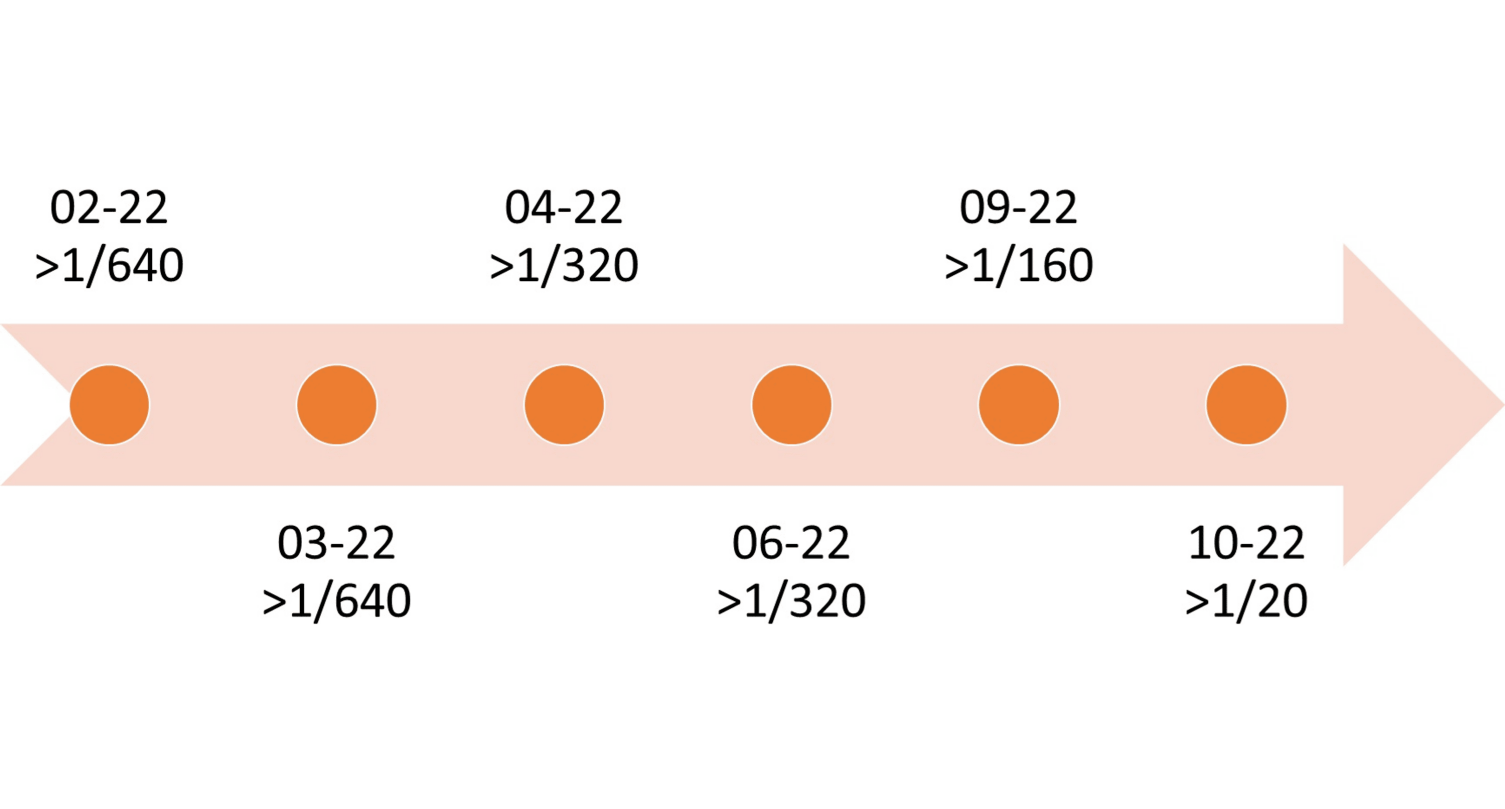
Evolution of the patient's Wright reaction. Antibody titers considered significant (higher than 1/160 or 1/320, in endemic areas) generally only appear after the second week. In this patient, dosing begins three weeks after the start of the clinic. The decrease in the title reflects the favorable evolution with the established therapy.

On February 16, 2022, the patient went to the Emergency Department due to the worsening of her condition with chest pain and palpitations. Physical examination showed a hypertensive blood pressure profile, tachycardia, and a normal body temperature. She was admitted to the Intensive Care Unit due to the development of complications, namely severe type I acute respiratory failure, *Brucella spp.* endocarditis with splenic and renal embolization, acute renal failure, myocarditis, and heart failure with reduced left ventricular ejection fraction (LVEF), with the diagnosis of brucellosis with systemic dissemination being assumed. During hospitalization, the Wright reaction was performed (the evolution of which is shown in Figure 1). Additionally, anti-brucella antibodies were determined (Table 1, Figure 2), namely, Immunoglobulin M (IgM) and Immunoglobulin G (IgG), in order to monitor the evolution of the disease and the response to the therapy instituted.

**Table 1:** Dosage of anti-Brucella antibodies. This dosage was carried out three weeks after the start of the clinic. From the second week onwards, it is expected that the IgM titer begins to decrease and the IgG titer begins to increase, with a correlation between high IgG titers and active infection.

Anti-Brucella antibodies			
	Quantitative result	Qualitive results	Reference values
IgG	18.1	Positive	<0.9
IgM	0.5	Negative	<0.9

## Discussion

In the past, efforts have been made to eradicate brucellosis, by reinforcing the education of professionals and consumers. However, more than 500,000 infections occur in humans each year [1]. Complications of brucellosis are rare but include subacute bacterial endocarditis, neurobrucellosis, orchitis, cholecystitis, hepatic suppuration, and osteomyelitis [10]. Mortality is around 6%, with higher figures being considered due to the disabling nature of the disease and its financial burden [9].

The clinical case presented relates to a patient with an episode of dairy product consumption, which is the most described form of infection due to brucellosis in Portugal, as mentioned in the ECDPC report [2]. In this context, a brief reference is made to the time when there were 80 new cases per year, to note that the numbers presented are currently low, largely due to a decrease in laboratory studies that allow its diagnosis. In this sense, and in accordance with the ECDPC report, diagnostic tests should be carried out as screening, within the scope of occupational medicine, in professionals who work with animals and, above all, in workers who have contact with sick animals [2].

In the literature, the possibility of performing molecular biology tests to diagnose brucellosis is discussed, as they can aid in performing a quick diagnosis; only a few hours are needed, with high sensitivity and specificity. However, in addition to being more expensive, a careful interpretation is required. A positive PCR test may not necessarily indicate an active infection, instead, it could be deoxyribonucleic acid (DNA) from dead organisms in recovered patients. The commercial molecular biology tests available for the diagnosis of brucellosis are still limited and there are still few published comparative studies evaluating the performance of the different commercial molecular tests and protocols developed by research laboratories [12].

For this reason, health and veterinary professionals must be instructed on the use of personal protective equipment when handling viscera and animal parts. Therefore, reinforcing the education of professionals and consumers in the district of Bragança is an essential measure, not forgetting the relevance of raising public awareness of the importance of serological screening.

Activities should be restricted in acute cases of brucellosis and bed rest is recommended during febrile episodes. Severe skeletal muscle pain, especially above the spine, may require analgesia. Even with antibiotic treatment, around 5 to 15% of patients experience general relapse, so all patients must be clinically monitored and with repeated serological titers for one year. Brucella endocarditis usually requires surgery in addition to antimicrobial treatment [10,11]. In the case presented, the patient, despite targeted treatment, developed endocarditis, as a serious complication, without the need for surgical treatment, but with the need for admission to the ICU.

There is no human vaccine and the use of animal vaccines in humans can cause infection. Immunity after human infection is short-lived, approximately two years [11].

## Conclusions

Brucellosis is a disease that is “neglected” due to its low apparent incidence, both in Portugal and worldwide, despite being one of the three most frequent zoonoses in our country and, especially, in the Trás-os-Montes region. Both the non-specific clinical presentation, as well as the risks, the existing comorbidities, and the presence of an aging population, should be considered when screening for this disease, especially at an occupational level. Cultural, serological, and imaging studies should be performed whenever advised, to identify associated complications, which will be part of the disease investigation protocol.

This clinical case provided an opportunity to highlight an endemic disease that seems somewhat forgotten today. In addition, it serves as a reminder of the importance of tests that were once routine in the past and continue to be essential for diagnosis and monitoring treatment. We also highlight the need to increase the literacy of agricultural and livestock professionals about this disease. Finally, we highlight the preventive role that public health teams can play in terms of raising awareness, promoting health, and implementing collective and individual measures for the early diagnosis of this disease, in order to reduce its comorbidities.

## References

[1] Hayoun MA, Muco E, Shorman M (2023). Brucellosis. StatPearls.

[2] (2023). Brucellosis - annual epidemiological report for 2020. for.

[3] Mendes A, Gomes B, Sousa L (2020). Brucellosis: a rapid risk assessment by a regional outbreak team and its coordinated response with the Directorate-General for Food and Veterinary, North region of Portugal, 2019. Zoonoses Public Health.

[4] (2024). National Animal Feed Production Report-2022, Directorate-General for Food and Veterinary Medicine, Government of Portugal (In Portuguese). https://www.dgav.pt/wp-content/uploads/2024/03/DGAV_RELATORIO-2022-PRODUCAO-NACIONAL-FINAL-19.03.24.pdf.

[5] Pappas G, Akritidis N, Bosilkovski M, Tsianos E (2005). Brucellosis. N Engl J Med.

[6] Pessegueiro P, Barata C, Correia J (2003). Brucellosis - a systematic revision (Article in Portuguese). Medicina Interna.

[7] Bosilkovski M, Krteva L, Dimzova M, Vidinic I, Sopova Z, Spasovska K (2010). Human brucellosis in Macedonia - 10 years of clinical experience in endemic region. Croat Med J.

[8] Bosilkovski M (2024). Brucellosis: epidemiology, microbiology, clinical manifestations and diagnosis. UpToDate.

[9] Despacho n.º 1150/2021 (2023). Order n.º1150/2021 (In Portuguese). Diário da República.

[10] Al-Tawfiq JA (2008). Therapeutic options for human brucellosis. Expert Rev Anti Infect Ther.

[11] Bush ML (2024). Brucellosis. MSD Manual. Reviewed/Revised Jun.

[12] Di Bonaventura G, Angeletti S, Ianni A, Petitti T, Gherardi G (2021). Microbiological laboratory diagnosis of human brucellosis: an overview. Pathogens.

